# RNA-seq Analysis of *Nepenthes ampullaria*

**DOI:** 10.3389/fpls.2015.01229

**Published:** 2016-01-11

**Authors:** Wan-Nor-Adibah Wan Zakaria, Kok-Keong Loke, Muhammad-Mu'izzuddin Zulkapli, Faris-'Imadi Mohd Salleh, Hoe-Han Goh, Normah Mohd Noor

**Affiliations:** Plant Functional Genomics Research Group, Institute of Systems Biology, Universiti Kebangsaan MalaysiaSelangor, Malaysia

**Keywords:** carnivorous plant, detritivore, digestive enzyme, illumina sequencing, transcriptome

## Introduction

Pitcher plant, *Nepenthes* spp., is one of the carnivorous plants, which produces unique pitcher structure from the tip of leaf tendril for trapping and digestion of insect prey to acquire nutrients at habitats that are deprived of nitrogen. *Nepenthes ampullaria* is special when compared with other pitcher plants as it has evolved a detritivore habit for the acquisition of nutrients from leaf litter instead of solely dependent on insects (Moran et al., 2003; Pavlovič et al., 2011). The morphology of pitcher changes upon opening in preparation for nutrient absorption (Owen et al., 1999). Furthermore, the efficiency of pitcher trapping increases from day 3 to day 6 after opening (Bauer et al., 2009). Hence, it is of interest to investigate the maturation of pitcher in the first 3 days of opening. To study the pitcher fluid composition and its gene expression changes over time, we performed the first transcriptome analysis of *N. ampullaria* for comparison with *N. ventrata* (Wan Zakaria et al., 2016). Raw reads of the transcriptome assembly project have been deposited to SRA database with the accession numbers SRX1400303 (Day 0 control), SRX1400308 (Day 3 control), and SRX1400311 (day 3 depleted).

## Value of the data

- *Nepenthes* spp. plants are one of the passive carnivorous genera that lack molecular genetics information. This hinders new protein discovery through proteomic approach to understand carnivory trait in pitcher plants.
- These data are important for the identification of unique digestive enzymes and aspartic proteinases from pitcher plant. This will improve our understanding of the evolutionary history of this carnivorous plant family (Pavloviè, 2012).
- Gene expression study of pitcher development can also provide insights on the regulation of digestive enzyme secretion into pitcher fluid.

## Data

Transcriptome profile of *N. ampullaria* was generated from the polyA-enriched cDNA libraries prepared from total RNA extracted from its pitcher. The short reads were filtered, processed, assembled, and analyzed as described in the next section. Raw data for this project were deposited at SRA database with the accession numbers SRX1400303 (http://www.ncbi.nlm.nih.gov/sra/SRX1400303) for day 0 control, SRX1400308 (http://www.ncbi.nlm.nih.gov/sra/SRX1400308) for day 3 longevity experiment, and SRX1400311 (http://www.ncbi.nlm.nih.gov/sra/SRX1400311) for day 3 fluid protein depletion experiment. Assembled transcriptome fasta sequences can be accessed at http://gohlab.researchfrontier.org/public-datasets/Nepenthes-ampullaria-Trinity-gohlab.fasta.

## Experimental design, materials, and methods

### Plant materials

*N. ampullaria* pitcher plants were grown under shady environment in the nursery of experimental plot (2°55′09.0″N 101°47′04.8″E) from Jan 2014 to Apr 2015 at Universiti Kebangsaan Malaysia, Bangi, Malaysia. Individual whole pitchers were collected in the morning and frozen in liquid nitrogen before storing at −80°C for further use. This study did not involve endangered plants from the wild.

Three different pitcher samples were collected, namely day 0 control, day 3 control, and day 3 protein depletion. Day 0 control sample was collected within 24 h of pitcher opening. For longevity experiment, to understand the effect of time after pitcher opening for gene expression, day 0 pitcher was sealed by parafilm and collected after 3 days. Parafilm was used to ensure that the pitcher fluid is not contaminated by any foreign bodies. For protein depletion experiment, pitcher fluids were syringe filtered through 0.22 μm PVDF membrane followed by ultrafiltration at molecular weight cutoff (MWCO) of 10,000 using Microsep Advance device (PALL, USA). The pitchers were then replenished by the filtrate with depleted protein bigger than 10 kDa, sealed by parafilm, and collected after 3 days.

### Total RNA extraction and quality control, library preparation, and RNA-seq

For RNA works, RNAs from all samples were extracted using modified method of CTAB (Kim and Hamada, 2005). Quantity and integrity of the extracted total RNA were determined using NanoDrop (Thermo Fisher Scientific Inc., USA) and Agilent 2100 bioanalyzer (Agilent Technologies, USA), respectively, to be RIN > 8.

One pitcher sample for each treatment of *N. ampullaria* was sequenced individually using the Illumina HiSeq 2500 sequencing platform. Paired end reads of 125 bp were generated through the standard polyA-enriched library preparation protocol implemented by Macrogen, South Korea.

### Transcriptome *de novo* assembly

Raw reads from all the three data sets were filtered to remove the adapter sequences with sequence pre-processing tool, Trimmomatic (Bolger et al., 2014). High quality Illumina raw reads with phred score ≥25 were kept for assembly. *De novo* assembly of these processed reads was performed using Trinity (v2.0.6) (Haas et al., 2013) with trimmomatic flag and default k-mer size (25) as recommended by Trinity. Assembly statistics were generated via utility script TrinityStats.pl. Table 1 shows the statistics of the transcriptome assembly. The assembled transcriptome fasta sequences can be used for gene discovery, further study on the pitcher physiology, and comparative transcriptome analysis with other *Nepenthes* species.

**Table 1:** **Statistics of ***N. ampullaria*** assembly**.

**Attributes**	**Value**
**PRE-ASSEMBLY**	
Total raw reads	141,483,256
Total processed reads	134,043,179
**POST ASSEMBLY**	
Number of unigenes	158,757
Number of unique transcripts	202,322
N50 (bp)	1,066
Size range (bp)	224–11,748

## Author contributions

WNAWZ: conducted the experiment; KKL, MMZ, FIMS, HHG: performed analysis on the data; HHG, NMN: conceived the project and acquired funding; WNAWZ, KKL, HHG: wrote the manuscript.

### Conflict of interest statement

The authors declare that the research was conducted in the absence of any commercial or financial relationships that could be construed as a potential conflict of interest. The reviewer Axel Fischer and handling Editor Dirk Walther declared their shared affiliation, and the handling Editor states that, nevertheless, the process met the standards of a fair and objective review.
